# Women Oncologists’ Perceptions and Factors Associated With Decisions to Pursue Academic vs Nonacademic Careers in Oncology

**DOI:** 10.1001/jamanetworkopen.2021.41344

**Published:** 2021-12-30

**Authors:** Emily C. Merfeld, Grace C. Blitzer, Aleksandra Kuczmarska-Haas, Susan C. Pitt, Fumiko Chino, Trang Le, Wendy A. Allen-Rhoades, Suzanne Cole, Ariela L. Marshall, Molly Carnes, Reshma Jagsi, Narjust Duma

**Affiliations:** 1 Department of Human Oncology, University of Wisconsin Hospital and Clinics, Madison; 2 Department of Surgery, University of Wisconsin Hospital and Clinics, Madison; 3 Department of Radiation Oncology, Memorial Sloan Kettering Cancer Center, New York, New York; 4 Department of Biostatistics, University of Wisconsin-Madison, Madison; 5 Department of Pediatric Hematology/Oncology, Mayo Clinic, Rochester, Minnesota; 6 Department of Hematology/Oncology, University of Texas Southwestern Medical Center, Dallas; 7 Department of Hematology/Oncology, Mayo Clinic, Rochester, Minnesota; 8 Department of Medicine, University of Wisconsin School of Medicine and Public Health, Madison; 9 Department of Radiation Oncology, University of Michigan, Ann Arbor; 10 Department of Medical Oncology, University of Wisconsin Hospital and Clinics, Madison; 11 Department of Medical Oncology, Dana-Farber Cancer Institute, Boston, Massachusetts

## Abstract

**Importance:**

Women outnumber men in US medical school enrollment, but they represent less than 40% of academic oncology faculty.

**Objective:**

To identify the key factors associated with female oncologists’ decision to pursue academic or nonacademic oncology practice and to characterize their perceptions about their current career.

**Design, Setting, and Participants:**

This cross-sectional survey study was distributed through email and social media to female physicians in academic and nonacademic oncology practice in the United States. The survey was open for 3 months, from August 1 to October 31, 2020.

**Main Outcomes and Measures:**

No single primary study outcome was established because of the cross-sectional nature of the survey. Data were collected anonymously and analyzed using *t* tests for continuous variables and χ^2^ tests for categorical variables.

**Results:**

Among the 667 female respondents, 422 (63.2%) identified as academic oncologists and 245 (36.8%) identified as nonacademic oncologists. Approximately 25% of respondents reported that their spouse or partner (156 [23.5%]) and/or family (176 [26.4%]) extremely or moderately affected their decision to pursue academic practice. Academic oncologists perceived the biggest sacrifice of pursuing academics to be time with loved ones (181 [42.9%]). Nonacademic oncologists perceived the biggest sacrifice of pursuing academics to be pressure for academic promotion (102 [41.6%]). Respondents had different perceptions of how their gender affected their ability to obtain a chosen job, with 116 academic oncologists (27.6%) and 101 nonacademic oncologists (41.2%) reporting a positive or somewhat positive impact (*P* = .001). More than half of the women surveyed (54.6% academic oncologists [230]; 50.6% nonacademic oncologists [123]; *P* = .61) believed they were less likely to be promoted compared with male colleagues. Academic and nonacademic oncologists reported rarely or never having a sense of belonging in their work environment (33 [7.9%] and 5 [2.0%]; *P* < .001). Most respondents reported that they would choose the same career path again (301 academic oncologists [71.3%]; 168 nonacademic oncologists [68.6%]); however, 92 academic oncologists (21.9%) reported they were likely to pursue a career outside of academic oncology in the next 5 years.

**Conclusions and Relevance:**

This survey study found that a spouse or partner and/or family were factors in the career choice of both academic and nonacademic oncologists and that female gender was largely perceived to adversely affect job promotion. Given that more than 20% of female academic oncologists were considering leaving academia, gender inequality is at high risk of continuing if the culture is not addressed.

## Key Points

**Question:**

What factors are associated with female oncologists’ decision to pursue academic or nonacademic practice?

**Findings:**

In this survey study of 667 female oncologists in the US, a spouse or partner and/or family were factors in the career choice of both academic and nonacademic oncologists. Most respondents perceived their gender to adversely affect job promotion, and more than 20% of respondents were considering leaving academia.

**Meaning:**

The findings suggest that gender inequality in academic oncology will continue if the culture is not addressed.

## Teaser

This survey study examines the role of family, personal priorities, and sense of professional belonging and fulfillment in career building or planning among female oncologists.

## Introduction

Underrepresentation of women in academic oncology is a growing concern. Enrollment of women in US medical schools has surpassed that of men, and 45.0% of hematology/oncology fellows, 30.3% of radiation oncology residents, and 70.5% of pediatric hematology/oncology fellows are women.^1^ However, women represent only 35.9% of academic oncology faculty, including 37.1% in hematology/oncology, 30.7% in radiation oncology, 38.8% in surgical oncology, and approximately 50% in pediatric hematology/oncology.^2,3,4^ Women are particularly underrepresented among the leadership in oncology specialties, occupying 31.4% of the chair positions in medical oncology, 17.4% in radiation oncology, and 11.1% in surgical oncology.^2^ Of the most recent leadership roles in professional oncology associations, 3 of the 25 presidents of the American Society for Radiation Oncology, 7 of the 25 presidents of the American Society of Clinical Oncology, 4 of the 25 presidents of the Society of Surgical Oncology, 4 of the 17 presidents of the American Society of Pediatric Hematology/Oncology, and 0 of the 5 directors of the National Cancer Institute were women.^5,6,7,8,9^ Data suggest that, although women may be more likely to pursue careers in academic medicine and may have similar research productivity compared with men, they also leave academia at a higher rate and are less likely to be promoted or appointed to leadership roles.^10,11,12^

Inequality in gender representation within academic oncology is pronounced. Yet, there is a paucity of data regarding the motivations of female physicians and scientists to build a career in academic oncology. The primary objective of this survey study was to identify the key factors associated with female oncologists’ decision to pursue academic or nonacademic oncology practice and to characterize their perceptions about their current career.

## Methods

This cross-sectional survey was approved by University of Wisconsin Institutional Review Board and distributed to self-identifying female oncologists in the United States. Written consent, in the form of agreement to a statement, was obtained from all survey participants before initiating the survey. We followed the American Association for Public Opinion Research (AAPOR) reporting guideline.

We developed the survey according to feedback from a multidisciplinary panel of experts in gender inequality in academia. The survey used branching logic to collect information on demographic and other factors that affect career choice. The survey was distributed by email using professional society lists and social media using Twitter and female physician groups on Facebook. A $5 coffee gift card was offered as an incentive for participation. The survey was open for 3 months, from August 1 to October 31, 2020.

All results were validated using screening methods to ensure that responses came from female oncologists. Screening methods involved discarding all responses with unreasonable answers (eg, work hours that added up to more than 168 hours/week). Responses were considered valid if they met multiple criteria, including use of institutional email and an email address bearing the name of a person who was found to be a practicing oncologist. Data were collected anonymously through SurveyMonkey (Momentive).

Female oncologists were asked to identify their current oncology practice, whether academic or nonacademic. Academic oncology practice was defined as clinical practice in a medical center that is dedicated to scholarly activities, including teaching and research, as well as patient care often in conjunction with a university or medical school. Nonacademic oncology practice was described as hospital-based practice, community clinical practice, hybrid practice, government employment, or industry employment (eg, in biotechnology and pharmaceutical fields), among other types. Participants self-identified their race and ethnicity as African American, Asian American, Hispanic, Indian American, Middle Eastern, White, or other (which was specified by the participant).

### Statistical Analysis

Demographic factors and factors of interest (ie, impact of spouse or partner and/or family, perception of sacrifices and priorities, and professional belonging and fulfillment) were summarized in mean (SD) or count (percentage) in both academic and nonacademic practice groups. Differences in these factors between the groups were analyzed using an unpaired, 2-tailed *t* test for continuous variables and the χ^2^ test for categorical variables. A 2-sided *P* < .05 was considered statistically significant. All analyses were conducted in R, version 4.0.5 (R Foundation for Statistical Computing).

## Results

A total of 667 female oncologists completed the survey; the demographic characteristics of respondents are summarized in the Table. Of the respondents, 422 women (63.2%) identified as academic oncologists and 245 (36.8%) identified as nonacademic oncologists. Practice settings of nonacademic oncologists are summarized in eTable 1 in the Supplement. Among the participants, 302 (45.3%) specialized in medical oncology/hematology, 173 (25.9%) in radiation oncology, 88 (13.2%) in pediatric hematology/oncology, 56 (8.4%) in surgical oncology, and 48 (7.2%) in other specialties. In terms of marital status, 560 respondents (84.0%) were married or had a partner and 109 (16.3%) were single, divorced, or widowed. Of those who were married or with partners, 507 (90.5%) reported having a spouse or partner who worked and 369 (65.9%) reported having a spouse or partner who worked full-time. In terms of children, 498 respondents (74.6%) had children, either before or during medical school (61 [12.2%]), during residency or fellowship (237 [47.6%]), or after training (200 [40.2%]). There was no significant difference in the timing of having children between the academic and nonacademic practice groups.

**Table.** Demographic Characteristics of Survey Respondents

Characteristic	Total, No. (%)	Oncology practice, No. (%)		*P* value
		Academic	Nonacademic	
No. of respondents	667 (100)	422 (63.2)	245 (36.8)	
Working status				
Full-time	611 (92.0)	400 (95.5)	211 (86.1)	<.001
Part-time	45 (6.8)	18 (4.3)	27 (11.0)	
Not working	8 (1.2)	1 (0.2)	7 (2.9)	
Year graduated from training				
2020-2016	190 (28.5)	138 (32.7)	52 (21.2)	.001
2015-2011	180 (27.0)	111 (26.3)	69 (28.2)	
2010-2006	133 (19.9)	85 (20.1)	48 (19.6)	
2005-2001	55 (8.2)	37 (8.8)	18 (7.3)	
2000-1991	68 (10.2)	34 (8.1)	34 (13.9)	
1990-earlier	41 (6.1)	17 (4.0)	24 (9.8)	
Age, y				
<30	8 (1.2)	4 (1.0)	4 (1.6)	.02
30-39	277 (41.6)	181 (43.0)	96 (39.2)	
40-49	245 (36.8)	167 (39.7)	78 (31.8)	
50-59	83 (12.5)	41 (9.7)	42 (17.1)	
60-69	45 (6.8)	25 (5.9)	20 (8.2)	
≥70	8 (1.2)	3 (0.7)	5 (2.0)	
Time at current job, y				
0-1	89 (13.4)	59 (14.1)	30 (12.3)	.76
>1-3	136 (20.5)	89 (21.2)	47 (19.3)	
>3-10	268 (40.4)	168 (40.1)	100 (41.0)	
>10	170 (25.6)	103 (24.6)	67 (27.5)	
Race and ethnicity^a^^,^^b^				
African American	45 (6.7)	32 (7.6)	13 (5.3)	.86
Asian American	159 (23.8)	97 (23.2)	62 (25.3)	
Hispanic	39 (5.8)	24 (5.7)	15 (6.1)	
Indian American	6 (0.9)	3 (0.7)	3 (1.2)	
Middle Eastern	10 (1.5)	7 (1.7)	3 (1.2)	
White	420 (63.0)	266 (63.0)	154 (62.9)	
Other^c^	6 (0.9)	4 (0.9)	2 (0.8)	
Specialty				
Medical oncology/hematology	302 (45.3)	182 (43.1)	120 (49.0)	<.001
Pediatric oncology/hematology	88 (13.2)	73 (17.3)	15 (6.1)	
Radiation oncology	173 (25.9)	84 (19.9)	89 (36.3)	
Surgical oncology	56 (8.4)	45 (10.7)	11 (4.5)	
Other	48 (7.2)	38 (9.0)	10 (4.1)	
Marital status^a^				
Single	80 (12.0)	51 (12.2)	29 (11.8)	.66
Married or with partner	548 (82.2)	348 (82.5)	200 (81.6)	
Divorced	22 (3.3)	12 (2.9)	10 (4.1)	
Widowed	7 (1.0)	3 (0.7)	4 (1.6)	
Other partnership^d^	12 (1.8)	9 (2.1)	3 (1.2)	
Children				
0	167 (25.1)	105 (25.0)	62 (25.3)	.90
1	140 (21.1)	90 (21.4)	50 (20.4)	
2-3	341 (51.2)	215 (51.2)	126 (51.4)	
≥4	17 (2.6)	10 (2.4)	7 (2.9)	

^a^ Multiple answers were allowed.

^b^ Race and ethnicity were self-identified in the survey by participants.

^c^ Other race and ethnicity were specified by participants.

^d^ Other partnership was specified by participants.

### Role of Spouse or Partner and/or Family

Approximately 25% of participants reported that their spouse or partner (156 [23.5%]) and family (176 [26.4%]) extremely or moderately affected their decision to pursue academic practice. The mean (SD) percentage of household tasks performed was 55.1% (20.3%) for academic oncologists and 55.2% (19.5%) for nonacademic oncologists. More than 99% of respondents outsourced at least 1 household or family task. No significant difference in the mean number of household or family tasks outsourced (2.2 tasks; *P* = .75) was found between oncologists in academic and nonacademic practice.

### Perception of Sacrifices and Priorities

**Figure 1.**
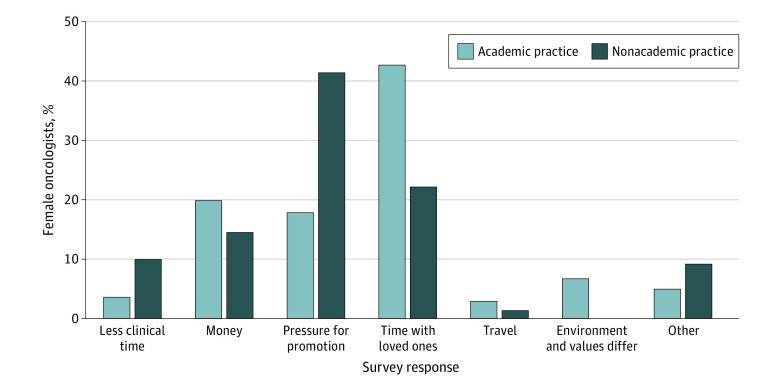
Perceived Biggest Sacrifice of Pursuing Academic Oncology

Academic oncologists reported that the aspects of their career they most enjoyed were being a specialist within a specialized field (129 [30.6%]) and practicing in an academically rich environment (125 [29.6%]). Nonacademic oncologists reported they most enjoyed the ability to focus on clinical skills and time with patients (129 [52.7%]). Academic oncologists perceived that the biggest sacrifice of pursuing an academic practice was time with loved ones (181 [42.9%]), followed by money (85 [20.1%]) and pressure for academic promotion (76 [18.0%]). Nonacademic oncologists perceived that the biggest sacrifice of pursuing academic practice would be pressure for academic promotion (102 [41.6%]) followed by time with loved ones (55 [22.4%]) (Figure 1).

**Figure 2.**
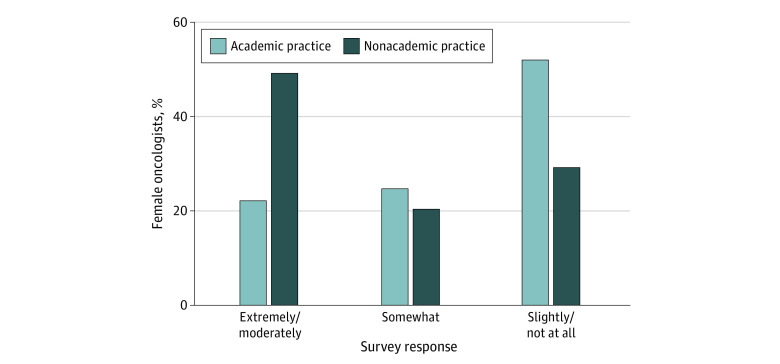
Perceived Role of Salary in Decision to Pursue Academic vs Nonacademic Oncology Practice

**Figure 3.**
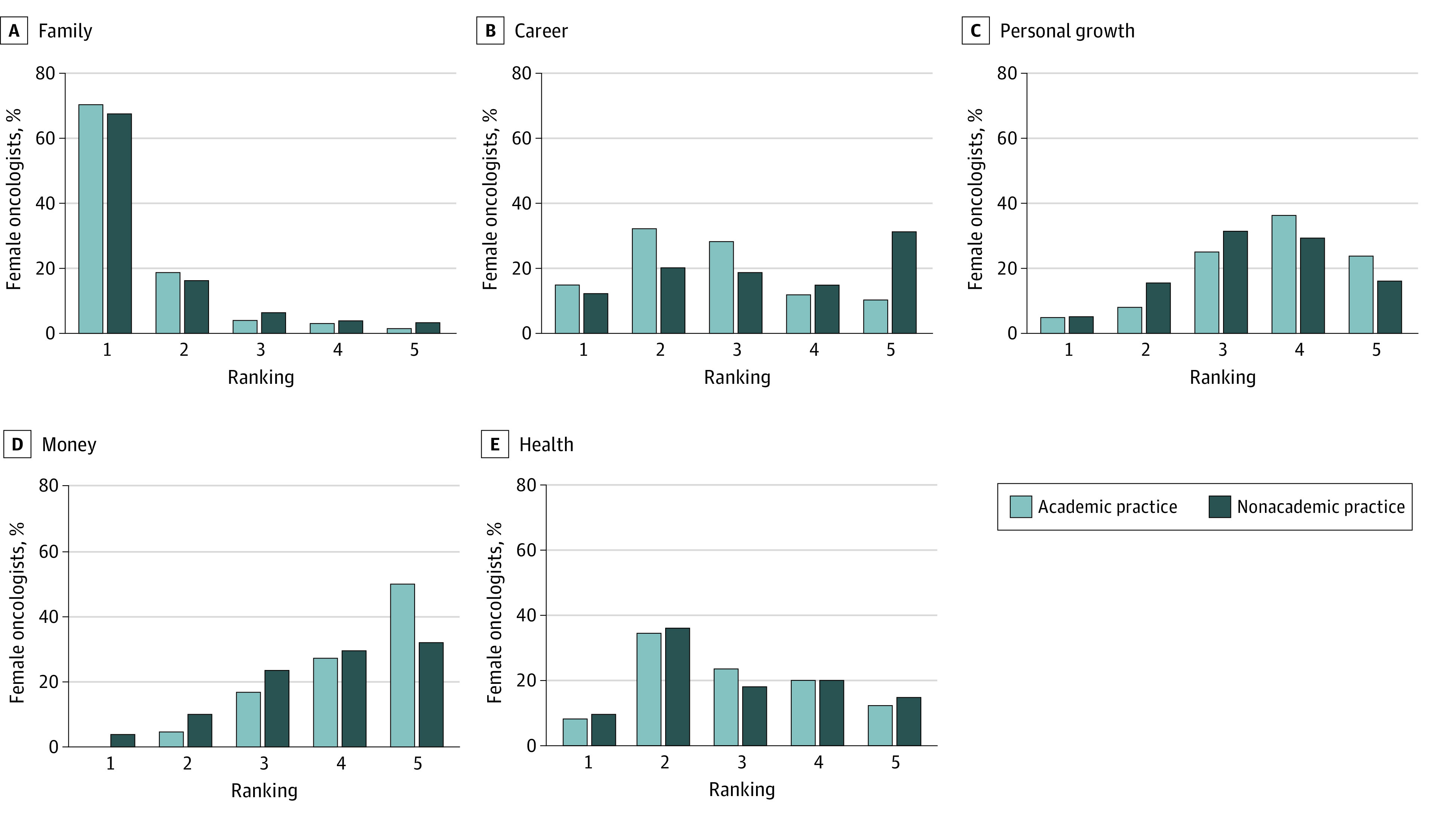
Ranking of Family, Career, Personal Growth, Money, and Health Priorities

There was no significant difference in the mean number of hours worked during the week (53 hours; *P* = .74) between respondents in the academic vs nonacademic practice groups. Academic oncologists compared with their nonacademic counterparts worked more hours on weekends (mean [SD], 10.9 [6.9] vs 8.8 [7.8] hours; *P* = .003) (eTable 2 in the Supplement). Salary extremely or moderately affected the decision of nonacademic oncologists to pursue a nonacademic career more so than it extremely or moderately affected the decision of academic oncologists to pursue an academic career (49.6% [121] vs 22.6% [95]; *P* < .001) (Figure 2). When asked to rank their priorities, most participants (464 [69.6%%]) ranked family as the number 1 priority. Academic oncologists ranked career as a higher priority, whereas nonacademic oncologists ranked personal growth and money as higher priorities (Figure 3).

### Professional Belonging and Fulfillment

**Figure 4.**
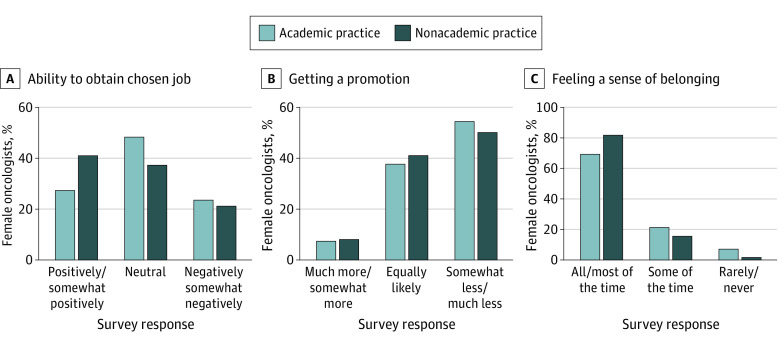
Perceived Implication of Female Gender for Obtaining a Chosen Job, Getting a Promotion, and Feeling a Sense of Belonging

Respondents in the academic vs nonacademic practice groups had significantly different perceptions on how their gender affected their ability to obtain a chosen job (Figure 4A). A larger proportion of nonacademic vs academic oncologists believed that their gender had a positive or somewhat positive impact (41.2% [101] vs 27.6% [116]; *P* = .001). A similar proportion of academic vs nonacademic oncologists believed that their gender had a negative or somewhat negative impact (23.8% [100] vs 21.2% [52]).

More than half of the respondents (54.6% academic oncologists [230] and 50.6% nonacademic oncologists [123]; *P* = .61) believed that they were less likely than their male colleagues to be promoted (Figure 4B). A total of 33 academic oncologists and 5 nonacademic oncologists reported rarely or never feeling a sense of belonging in their work environment (7.9% and 2.0%; *P* < .001) (Figure 4C). Most participants reported that they would choose the same career path again (301 academic oncologists [71.3%]; 168 nonacademic oncologists [68.6%]). However, 92 academic oncologists (21.9%) reported that they were likely or very likely to pursue a career outside the academic setting within the next 5 years. Among the 92 academic oncologists who were likely or very likely to leave academia, 26 (28.2%) stated they would switch to industry employment and 23 (25.0%) would switch to community clinical practice (eTable 3 in the Supplement).

## Discussion

Women are underrepresented in academic oncology. The reality that few female oncologists hold leadership roles propagates the problem of underrepresentation by reducing mentorship and sponsorship opportunities for young women in academic oncology.^13,14^ Multiple factors are likely involved in the choice of female oncologists to pursue a nonacademic career path or to switch from academia to private practice or industry. This study found that a spouse or partner and/or family were a factor in the career decision of female oncologists who chose to pursue academic or nonacademic practice, and the timing of having children and outsourcing of domestic duties were similar for women in both career paths. More than 50% of women in both academic and nonacademic practice perceived their gender to adversely affect their job promotion. Furthermore, 21.9% of academic oncologists reported that they were likely to leave academic oncology in the next 5 years.

One potential explanation for the underrepresentation of women in academic oncology is the social expectation for women compared with men to spend more time on domestic tasks and to be more invested in activities that are related to raising children, including child care.^15^ Multiple studies have found that female physicians spent more time on household and family tasks and were more likely to have a spouse or partner who worked full-time.^16,17^ However, the present study demonstrated no difference in the timing of having children or in the percentage of household tasks performed by female oncologists in both academic and nonacademic practice. This finding disputes the notion that competing commitments to domestic duties and career building lead women to favor nonacademic careers and that women believe nonacademic practice to be more family friendly than the academic setting. Furthermore, although we found that a spouse or partner and family were a substantial factor for pursuing an academic vs nonacademic career in 1 in 4 (25%) female oncologists, this frequency did not differ between academic and nonacademic oncologists. Partners or spouses and/or family did not seem to be a major factor in academic practice losing out to nonacademic practice in the recruitment and retention of female oncologists.

Nevertheless, 69.6% of oncologists ranked family as their number 1 priority, and 42.9% of academic oncologists perceived the biggest sacrifice to pursuing academic practice to be time with loved ones. As such, women in academic oncology reported working 2 more hours per weekend than their nonacademic counterparts. The perceived sacrifice of family time and the corresponding amount of work on the weekends are likely factors associated with burnout among female academic oncologists. Burnout affects all female oncologists and is estimated to be 20% to 60% higher among female than male physicians.^16^ This disparity may be secondary to the fact that burnout is commonly associated with work-life conflict, which has been shown to be more prevalent in women than men.^16,17,18^ Frustration with work-life balance is a key factor in female physicians’ decision to leave a career in academic medicine.^11^ Both nonacademic and academic oncologists worked a similar number of total weekly hours, and only 22.4% of nonacademic oncologists perceived time with loved ones to be the biggest sacrifice in pursuing academic oncology. Although work-life balance was a concern for academic oncologists and may be a factor in female oncologists leaving academia, survey data suggested that women in nonacademic practice faced similar challenges.

In this survey, academic oncologists ranked career higher on their list of priorities, whereas nonacademic oncologists ranked personal growth and money higher. The increased financial compensation in nonacademic oncology may play a large role in some women’s career decisions. Studies have reported that female academic physicians were paid a lower salary compared with male academic physicians, even after adjusting for age, experience, specialty, faculty rank, research productivity, and clinical revenue.^19,20^ In gynecologic oncology, a discrepancy in compensation between male and female oncologists was noted only in the academic setting.^21^

We found a clear dichotomy in the perception of academic and nonacademic oncologists on how their gender affected their ability to obtain a chosen job. More than 40% of nonacademic oncologists believed that their gender helped in obtaining a job compared with 27.6% of academic oncologists. In contrast, Banerjee et al^22^ found that the belief that women were less committed to their careers compared with men was more prevalent in the nonacademic than in the academic setting. This finding suggests that women in nonacademic oncology are becoming increasingly recognized as key members of the oncologic treatment team.

Most academic and nonacademic oncologists believed that their gender adversely affected their job promotion. This perception is confirmed by studies that found women were less likely than men to be promoted to associate professor, full professor, or department chair positions.^2,12^ This discrepancy has been confirmed specifically in the medical oncology, radiation oncology, and surgical oncology specialties.^2,14^ The perceived association of female gender with lack of promotion in academic oncology may be a factor in women opting for a career in a nonacademic setting.

Although the absolute proportion of women reporting a poor sense of belonging was low among both academic and nonacademic oncologists, this poor sense of belonging was significantly more common among academic oncologists. This finding was consistent with that in the study by Pololi et al,^23^ who found that women did not feel equally included in the academic environment and felt less confident about their career advancement compared with men. The lack of female role models in academic oncology and the poor representation of women in leadership positions are likely associated with the low self-confidence in career advancement and the internalization of stereotype threat among female physicians.^2,23^

Academic oncology remains at high risk for continued gender inequality if the culture is not addressed. Strategies for improving recruitment and retention of female academic oncology faculty members include reducing gender-based disparities in salary and promotion as well as developing a sense of belonging to ensure identity safety and minimize stereotype threat.^24^ Specifically, removing visible reminders that women are not welcome and training faculty members to provide performance feedback that is free of sexism are important next steps.^25,26^

We believe this survey provides a valuable understanding of the experiences and perceptions of female oncologists. In addition, the findings are hypothesis-generating given other data clearly demonstrating the underrepresentation of women in academic oncology. Future studies are warranted to further examine this study’s findings, including the association of gender perception with career length, and to compare the motivations behind the career choices and departure from academic oncology by women vs men.

### Limitations

This study has several limitations. First, gender was classified in a binary fashion (women vs men). Second, the survey was distributed mainly through email and social media, making the total denominator of female oncologists who were reached impossible to calculate. In addition, the use of social media posed a selection bias. However, social media in medicine is becoming widespread, with 72% of oncologists and oncology trainees in Canada using social media in 2016 and with Twitter increasingly used at annual oncology meetings.^27,28,29^ Basing an outreach strategy for survey research on social media has been done successfully in a previous survey.^30^ Nevertheless, the results of the current survey were likely most representative of the experiences of women with demographic characteristics similar to our respondents, such as younger age. Most of the participants in this survey (63.0%) self-identified as non-Hispanic White women. The small number of female oncologists who self-reported as having African American, Asian American, Hispanic, Indian American, Middle Eastern, or other race and ethnicity limited us from conducting an analysis of the association of race and ethnicity with career choice. Third, no men were included in the survey, making it impossible to ascertain whether the factors associated with career choice and perceptions about their current careers are unique to women. However, female physicians have been found to leave academic medicine at higher rates than male physicians.^31^

## Conclusions

This study found that, contrary to popular assumptions, a spouse or partner and/or family were not a major factor in female oncologists favoring nonacademic careers, because this factor was similarly important to both academic and nonacademic oncologists. Most female oncologists in both career paths perceived their gender to adversely affect job promotion; more nonacademic vs academic oncologists believed that their gender helped in obtaining their chosen job; more academic oncologists reported a poor sense of belonging in the workplace; and more than 20% of the academic oncologists in the current survey were considering leaving academia in the next 5 years. Academic oncology is at high risk for continued gender inequality if its culture is not addressed.

## Notes

Supplement. **eTable 1.** Practice Setting of Nonacademic Oncologists **eTable 2.** Hours Worked, Academic vs Nonacademic Oncologists **eTable 3.** Women Academic Oncologists’ Next Career Choice Upon Leaving Academics
