# Supplementary material for: Women Oncologists’ Perceptions and Factors Associated With Decisions to Pursue Academic vs Nonacademic Careers in Oncology
Source: JAMA Netw Open. 2021 Dec 30;4(12):e2141344. doi: 10.1001/jamanetworkopen.2021.41344 (PMC8719237; doi:10.1001/jamanetworkopen.2021.41344)
Supplement: Supplement. — eTable 1. Practice Setting of Nonacademic Oncologists eTable 2. Hours Worked, Academic vs Nonacademic Oncologists eTable 3. Women Academic Oncologists’ Next Career Choice Upon Leaving Academics [file jamanetwopen-e2141344-s001.pdf]

Supplementary Online Content

Merfeld EC, Blitzer GC, Kuczmarska-Haas A, et al. Women oncologists' perceptions and factors associated with decisions to pursue academic vs nonacademic careers in oncology. *JAMA Netw Open*. 2021;4(12):e2141344.
doi:10.1001/jamanetworkopen.2021.41344

eTable 1. Practice Setting of Nonacademic Oncologists

eTable 2. Hours Worked, Academic versus Nonacademic Oncologists

eTable 3. Women Academic Oncologists' Next Career Choice Upon Leaving Academics

This supplementary material has been provided by the authors to give readers additional information about their work.

© 2021 Merfeld EC et al. *JAMA Network Open.*

eTable 1. Practice Setting of Nonacademic Oncologists

	n (%)
**Hospital based**	107 (43.7%)
**Private practice**	79 (32.2%)
**Hybrid**	18 (7.3%)
**Government**	12 (4.9%)
**Industry**	17 (6.9%)
**Other**	12 (4.9%)

© 2021 Merfeld EC et al. *JAMA Network Open.*

eTable 2. Hours Worked, Academic versus Nonacademic Oncologists

	Academic oncologists	Nonacademic oncologists	p-value
**Weekday (hours/week)**	53.2 (17.7)	53.4 (20.0)	0.739
Clinical work	30.5 (16)	40.2 (17.5)	<0.001
Non-clinical work	22.7 (15.6)	13.2 (16.0)	<0.001
**Weekend (hours/week)**	10.9 (6.9)	8.8 (7.8)	0.003
Clinical work	4.8 (3.7)	5.2 (5.3)	0.275
Non-clinical work	6.1 (4.8)	3.6 (3.8)	<0.001

© 2021 Merfeld EC et al. *JAMA Network Open.*

eTable 3. Women Academic Oncologists' Next Career Choice Upon Leaving Academics

	n (%)
**Industry**	26 (28.2%)
**Consulting**	17 (18.5%)
**Community Practice**	23 (25%)
**Leave Medicine**	19 (20.7%)
**Other**	7 (7.6%)

© 2021 Merfeld EC et al. *JAMA Network Open.*
